# Surgery

**Published:** 1876-10

**Authors:** 


### VII. Surgery.

1. *Fracture of the Base of the Cranium.* Voltolini. (*Monatschr. für Ohrenheilk*, No. 1, 1876.)

The author concludes, from a review of published cases, that where the encephalo-rachidian fluid escapes — and especially when the result is favorable — the liquid does not proceed from the sub-arachnoid space, but from fracture of the labyrinth. If the liquid were cephalo-spinal there must have been necessarily a rupture of the dura-mater (a dangerous lesion of this organ), and the opening would remain patulous, permitting the escape of liquid for a considerable time. This cannot occur without the concomitant of serious symptoms of inflammation of the meninges, as in the first three cases reported. If a rupture of the dura-mater happens, there are two passages by which the liquid can reach the external auditory conduit, viz.: by the vault of the tympanic enclosure, the enclosure itself, and then through the membranum tympani; or the fissure may involve the promontory, when, if the arachnoid and dura-mater are both torn, the labyrinthine and encephalo-spinal liquids escape together.

We do not know the rapidity with which the labyrinthine liquid can be reproduced, so when a large amount of a liquid is discharged by the ear it cannot be determined whence it comes. Voltolini believes that the cases of Birkett, Heath and Morris, which resulted in a cure, were instances of labyrinthine fracture; and to prove that the labyrinth may become the site of a grave lesion without serious encephalic complication, he cites the cases of labyrinthine necrosis and otitis in which all alterations were noted as limited to situations external to the cranial cavity.

The practical inference is: always examine with care the condition of the sense of hearing in an injured individual.

2. *Traumatism by the bite of Horses.* Tillaux. (*La France Méd.*, No. 43.)

The author of the paper read by Tillaux (M. Gillette) before the Société

de Chirurgie had made sixty-six observations, in fifty of which the superior part of the body was injured by the bite of the horse. The animal is generally not content with seizing the part attacked between the jaws, he exercises more or less pressure upon it, or raises the individual from the earth, so as to tear and strip the tissues. We have then, generally, either pressure or tearing. Two ecchymotic arcs are characteristic of the bite by pressure.

The parts which are bitten and torn are not all alike. There may be produced a section as complete as if done by a cutting instrument. Thus, in one case, a horse tore off the finger of a soldier, after having made so clean a cut that the man was accused of self-mutilation.

The accidents caused by horse bite are of various sorts. In three cases tetanus occurred. In three others a curious fact was noticed: in consequence of contusion of the nerve in its canal, there resulted paralysis and permanent loss of power in the upper limb. Prognosis should be guarded in such cases, for from the cutaneous symptoms alone the injury to deeper parts cannot be determined.

The treatment consists in making free openings, but Gillette employs also the actual cautery. There is a question to be solved, whether the saliva of the horse does not contain a virulent principle.

3. *On the Treatment of In-growing Toe Nail.* BERTHERAUD. (*Gaz. Méd. de l' Algérie—Revue de Thér. Méd. Chir.*)

The author, after fully describing the disorder and discussing Dupuytren's sole remedy—complete avulsion of the nail—suggests the following procedure: The in-growing portion of the nail is first torn or cut away, according to the circumstances of each case, and then the fleshy part in which it was buried is covered with a disk of plaster, having a central perforation. Upon this central opening is placed a layer of Vienna paste, which thus destroys just enough of the inflamed surface, without causing too great a loss of substance. When the eschar falls it leaves an excavated wound, whose depression is increased later by the cicatricial retraction. When the nail grows again there is nothing to arrest its lateral expansion —it passes *over* the retracted tissues. The cure is perfect, and there is no relapse.

(A better, because it is a simpler, method has been devised, by Dr. Frank Hamilton, we believe, which accomplishes the same end. The nail is left undisturbed; but the point of a bistoury is passed along that part which is buried in the flesh, and, with a single sweep, all the soft tissue is removed beyond the nail border. Thereafter, the result is that given above. The cicatrix prevents the reformation of the granulating spongy mass, and the nail grows downward over it.—ED.)

4. *New Method of Staphylorrhaphy.* SCHOENBORN. (*Arch. f. Klin. Chir. XIX.—Centralbl. f. Chir.* 21, 1876.)

In order to remove the nasal sound of the voice which remains even after a successful uranoplasty and staphylorrhaphy, Sch. suggests the insertion, between the two halves of the soft palate, of a flap taken from the posterior wall of the pharynx. Sch. advises by the first operation to unite this pharyngeal flap with the soft palate, and after this union is established to close the fissure of the hard palate by a second operation. In regard to the formation of the pharyngeal flap he says: "After paring the edges of the cleft of the soft palate, one cuts out, with a scalpel having a long stem, an oblong flap in the posterior wall of the pharynx. The flap is cut from below upwards, with its basis below and its triangular-shaped apex as high up as possible, and is taken pretty thick; well dissected off from the subjacent structures, it is fastened to and between the halves of the cleft velum palati by means of sutures." Sch. has performed this operation on a girl aged seventeen years. Immediately after her recovery her speech was clear and easily intelligible; the nasal sound had not disappeared entirely yet, but it grew less from week to week. There was no difficulty in deglutition or in breathing through the nose.

